# Diphtheria: A Disease of the Liver (Extract)

**Published:** 1882-12

**Authors:** 


"DIPHTHERIA is a Disease of the Liver, according to Dr. Reiter (Squibb's *Ephemeris*). The liver does not destroy fibrin as it normally should. Dr. R. cures all cases by giving calomel in a scruple dose, followed by ten-grain doses every hour." What next?

